# Clinical and pathological factors influencing survival in a large cohort of triple-negative breast cancer patients

**DOI:** 10.1186/s12885-017-3969-y

**Published:** 2018-01-08

**Authors:** Silvana Anna Maria Urru, Silvano Gallus, Cristina Bosetti, Tiziana Moi, Ricardo Medda, Elisabetta Sollai, Alma Murgia, Francesca Sanges, Giovanna Pira, Alessandra Manca, Dolores Palmas, Matteo Floris, Anna Maria Asunis, Francesco Atzori, Ciriaco Carru, Maurizio D’Incalci, Massimo Ghiani, Vincenzo Marras, Daniela Onnis, Maria Cristina Santona, Giuseppina Sarobba, Enrichetta Valle, Luisa Canu, Sergio Cossu, Alessandro Bulfone, Paolo Cossu Rocca, Maria Rosaria De Miglio, Sandra Orrù

**Affiliations:** 1 Biomedicine Sector, Center for Advanced Studies, Research and Development in Sardinia (CRS4), Technology Park Polaris, Cagliari, Italy; 2 Department of Epidemiology, IRCCS - Istituto di Ricerche Farmacologiche Mario Negri, Via G. La Masa 19, 20156 Milan, Italy; 3 Department of Oncology, IRCCS-Istituto di Ricerche Farmacologiche “Mario Negri”, Milan, Italy; 4 Department of Pathology, “A. Businco” Oncologic Hospital, ASL, Cagliari, Cagliari Italy; 5 Department of Clinical and Experimental Medicine, University of Sassari, Sassari, Italy; 6 Department of Biomedical Sciences, University of Sassari, Sassari, Italy; 7 Department of Pathology, AOU, Sassari, Sassari Italy; 8 Department of Medical Oncology, “A. Businco” Oncologic Hospital, ASL, Cagliari, Cagliari Italy; 9 Department of Pathology, Brotzu Hospital, Cagliari, Italy; 10 Medical Oncology Unit, AOU, Cagliari, Italy; 11 Department of Pathology, ASL Nuoro, Nuoro, Italy; 12 Department of Medical Oncology, ASL Nuoro, Nuoro, Italy

**Keywords:** Clinicopathologic factors, Prognostic factors, Stage, Survival, Triple-negative breast cancer

## Abstract

**Background:**

To provide further information on the clinical and pathological prognostic factors in triple-negative breast cancer (TNBC), for which limited and inconsistent data are available.

**Methods:**

Pathological characteristics and clinical records of 841 TNBCs diagnosed between 1994 and 2015 in four major oncologic centers from Sardinia, Italy, were reviewed. Multivariate hazard ratios (HRs) for mortality and recurrence according to various clinicopathological factors were estimated using Cox proportional hazards models.

**Results:**

After a mean follow-up of 4.3 years, 275 (33.3%) TNBC patients had a progression of the disease and 170 (20.2%) died. After allowance for study center, age at diagnosis, and various clinicopathological factors, all components of the TNM staging system were identified as significant independent prognostic factors for TNBC mortality. The HRs were 3.13, 9.65, and 29.0, for stage II, III and IV, respectively, vs stage I. Necrosis and Ki-67 > 16% were also associated with increased mortality (HR: 1.61 and 1.99, respectively). Patients with tumor histotypes other than ductal invasive/lobular carcinomas had a more favorable prognosis (HR: 0.40 vs ductal invasive carcinoma). No significant associations with mortality were found for histologic grade, tumor infiltrating lymphocytes, and lymphovascular invasion. Among lymph node positive TNBCs, lymph node ratio appeared to be a stronger predictor of mortality than pathological lymph nodes stage (HR: 0.80 for pN3 vs pN1, and 3.05 for >0.65 vs <0.21 lymph node ratio), respectively. Consistent results were observed for cancer recurrence, except for Ki-67 and necrosis that were not found to be significant predictors for recurrence.

**Conclusions:**

This uniquely large study of TNBC patients provides further evidence that, besides tumor stage at diagnosis, lymph node ratio among lymph node positive tumors is an additional relevant predictor of survival and tumor recurrence, while Ki-67 seems to be predictive of mortality, but not of recurrence.

**Electronic supplementary material:**

The online version of this article (10.1186/s12885-017-3969-y) contains supplementary material, which is available to authorized users.

## Background

With an estimated 1.8 million new patients each year, breast cancer is the most common cancer in women worldwide [1]. In Italy, age-standardized (European standard) incidence and mortality rates in 2012 were 118/100,000 and 23/100,000, respectively, i.e., higher than those from other southern European countries [2].

Since 2005, triple-negative breast cancer (TNBC) identifies a specific subtype of breast cancer, characterized by the lack of expression of estrogen receptor (ER), progesterone receptor (PR), and human epidermal growth factor receptor 2 (HER2) [3]. TNBCs include a heterogeneous group of diseases which account for about 10–20% of all breast cancers and are more frequent among African American and Hispanic women, and in women with younger age, higher premenopausal body mass index, earlier age at menarche, and higher parity [3–5]. Moreover, they have higher expression of the Ki-67 antigen, higher mitotic index, and more frequent BRCA1 mutations. TNBCs are generally more aggressive than other breast neoplasms and have limited therapeutic options; therefore, they have usually a high risk of recurrence or death within 5 years since diagnosis [6].

Data on the clinical and pathological prognostic determinants for TNBC tumors are scanty and inconsistent and they generally derive from small hospital cancer registries including around a few hundreds of patients. TNM stage – including in particular the number of axillary lymph nodes involved – is one of the best-established prognostic factors for breast cancer, but its prognostic value in TNBCs, as in other intrinsic subtypes of breast cancer, is less clear [7, 8]. The ratio of positive lymph nodes on the total number of lymph nodes removed has been proposed as an additional and more accurate prognostic factor than the number of lymph nodes involved, although only a few studies have specifically evaluated its role in TNBC survival [9]. Although histologic grade has been shown to be a good predictor of survival for breast cancer, its prognostic role in TNBCs may be more limited given that most of these tumors are of high grade [10]. Furthermore, the findings on the prognostic value of the proliferation marker Ki-67 in TNBCs have been inconsistent [11]. Scantier data exist on tumor histotypes, tumor infiltrating lymphocytes (TIL), necrosis, and lymphovascular invasion (LVI) and survival from TNBC [12–15].

The primary aim of the study was therefore to provide further information on the clinical and pathological factors contributing to its prognosis of this subtype of breast cancer, i.e., survival or cancer progression, taking advantage of data from a uniquely large cohort of TNBC patients enrolled in Sardinia.

## Methods

Our study included 1152 women with a new, histologically confirmed diagnosis of TNBC, identified between 1994 and 2015 in Sardinia, an Italian region with around 1.68 million inhabitants and 1500 new breast cancer patients each year (incidence 127/100,000) [16]. TNBC cases were retrospectively selected through a complete review of surgical samples and medical records of breast cancer women treated in the main oncology hospitals of the region.

The study protocol was approved by the local research ethics committee of Sardinia Region (File number 224/CE/12). Informed consent was waived since patients’ information was collected during the routine clinical practice and patients were identified by anonymized investigator-generated code not linkable to their personal data.

### Immunohistochemical analysis

TNBC status was evaluated by immunohistochemistry using specific antibodies against monoclonal rabbit ER antibody, Clone SP1 (Neomarker) and monoclonal mouse anti-human PR antibody, Clone PgR 636 (Dako). ER and PgR expression was interpreted as positive if at least 1% immunostained tumor nuclei were detected in the sample, according with ASCO/CAP recommendations for immunohistochemical testing of hormone receptors in breast cancer [17]. HER2 protein expression was determined using FDA approved HercepTest™ (K5206 DAKO) and evaluated according to the manufacturer’s instructions. HER2 gene amplification was determined by ultra-View SISH Detection Kit (Ventana Medical Systems, Tucson, USA). Tumors were classified according to the 2013 ASCO/CAP recommendations [18]. Given that the study included patients diagnosed over almost 20 years in different hospital centers, all surgical specimens of TNBC patients were reviewed independently by three experienced pathologists to achieve a consensus on morphologic criteria and to standardize the results at the current guidelines recommendations for ER, PgR and HER2 immunohistochemistry [17].

### Baseline data

For each TNBC patient, personal and medical data were retrospectively collected from medical records and systematically integrated into a comprehensive database. The final database included patients’ information on socio-demographic factors, anthropometric characteristics, obstetric and gynecologic features, lifestyle habits, family history of breast and other cancers, and various comorbidities. Clinicopathological data of TNBC, including tumor site, histologic type and grade, TNM classification (tumor size, T, pathological lymph node status, pN, and distant metastases, M), TIL, necrosis, LVI, and expression of Ki-67 were also collected at cancer diagnosis. Tumor type was determined according to the UICC-WHO criteria [19] and tumor grade was established according to the Nottingham scheme [20]. Breast cancer TNM staging was defined according to the 7th edition of the American Joint Committee on Cancer criteria (AJCC) [21]. TILs were evaluated in the intra-epithelial compartment, in the stroma, and in the tumor periphery. LVI was defined as the presence of tumor emboli in peritumoral lymphatic spaces, capillary or postcapillary venules.

Lymph node ratio was defined as the number of positive lymph nodes divided by the number of lymph nodes evaluated. Lymph node ratio was then categorized according to validated cut-off points, i.e., <0.21, 0.21–0.65, and >0.65 [9].

### Follow-up data

During the study period, all clinical data of TNBC patients, including cancer treatments (surgery, radiotherapy and/or chemotherapy), TNBC recurrence, occurrence of other neoplasm(s), metastasis or death, were recorded. A review of all clinical charts allowed us to integrate information on cancer treatment and mortality into the database. Moreover, for each patient vital status was ascertained by enquiring the Sardinian registries of health. Since electronic cause-of-death certificates were available only for a small proportion of women (i.e., those dead at hospital or over most recent calendar years), this information was not used in our analyses. Cancer recurrence or occurrence of other neoplasms was assessed by the medical oncologists.

Overall survival (OS) was defined as the time between the date at diagnosis and the date of death (from any cause); disease-free survival (DFS) was defined as the time from the date at diagnosis to the date of local recurrence of TNBC, occurrence of other primary cancers, clinical metastatic diseases, death, or last follow-up visit, whichever occurred first. For the analysis of recurrence or DFS, 16 patients with missing information on cancer recurrence or with metastasis at baseline were excluded.

### Statistical analysis

The multivariate hazard ratios (HRs) for mortality or recurrence according to various clinicopathological characteristics, and the corresponding 95% confidence intervals (CIs), were estimated using Cox proportional hazards models. The models were adjusted for study center and age at diagnosis. HRs further adjusted for clinicopathological factors found to be significantly associated (*p* < 0.05) to mortality or recurrence in the center and age-adjusted analyses were also estimated. In multivariable models, a complete cases analysis was performed; as a sensitivity analysis, the missing indicator method was also used. The Cox proportional hazards assumptions for each covariate were checked graphically and using the Schoenfeld’s test. Kaplan-Meier method and Log-Rank test were used to describe OS and DFS according to various factors of interest. All the analyses well performed using the SAS software version 9.4 (SAS Institute Inc., Cary, NC, USA).

## Results

Among a total of 1152 TNBC patients, 311 (27%) did not have information on vital status at follow-up and had a high proportion of missing for most variables considered. Therefore, overall 841 TNBC patients were included in the analysis of mortality or OS and 825 for the analysis of recurrence or DFS. Of these, 275 (33.3%) had a progression of the disease (i.e., either cancer recurrence, metastasis, or death) and 170 (20.2%) died after a mean follow up of 4.3 years (standard deviation, SD, 3.7). Five-year overall survival (OS) and disease-free survival (DFS) were 75.0% and 63.6%, respectively.

Table 1 shows the distribution of TNBC patients, according to selected patients’ characteristics. Mean age of TNBC patients was 55.8 (SD 13.5); 46.5% of patients had a diagnosis before age 50. Overall, 42.3% of patients had menarche at age 12–13 years. Almost two-thirds of patients (67.4%) have had at least one child and 30.3% of patients were in pre-menopause. With reference to clinical and pathological characteristics of TNBCs at diagnosis, 74.3% of TNBCs were invasive ductal, 7.7% lobular, 3.4% medullary, 2.4% apocrine, 1.7% pleomorphic, 1.4% metaplastic carcinoma, and 3.4% were other carcinomas. 1.3% of TNBCs were of grade 1, while the large majority of TNBCs (71.3%) were grade 3. Overall, 36.7% of TNBCs were classified as T1, 43.3% as T2, 6.5% as T3, and 5.1% as T4. According to number of lymph nodes involved, 52.2% were classified as pN0, 22.0% as pN1, 10.5% as pN2, and 5.6% as pN3. At the time of diagnosis, 10 of TNBC patients (1.2%) presented metastasis, the site of involvement of metastatic disease being mostly liver (80%), followed by bone (50%) and lung (30%), and 50% of patients had a metastasis at one single site (data not shown). Overall, 25.1% of TNBCs were stage I, 42.7% stage II, 19.1% stage III, and 1.2% stage IV (Table 1). TILs were present in more than one third (33.7%) of TNBCs, LVI in 23.0% of tumors, and necrosis in 35.8%. Ki-67 protein was highly expressed (i.e., ≥46%) in 44.7% of TNBCs (median value 40%, range 0–95%). 52.0% of women had a quadrantectomy and 37.9% had a mastectomy; of those who received a quadrantectomy, 94.1% received radiotherapy, while of those who ad a mastectomy, 7.5 received radiotherapy; finally, 4.0% of women had neoadjuvant chemotherapy only, 64.8% adjuvant chemotherapy only, and 8.7% received both treatments.

**Table 1:** Characteristics of 841 triple-negative breast cancer (TNBC) patients. Sardinia, Italy 1994–2015

	TNBC patients	
	N	%
Calendar period at diagnosis		
1994–2004	289	34.4
2005–2009	294	34.9
2010–2015	258	30.1
Age at diagnosis (years)		
< 45	190	22.6
45–54	201	23.9
55–64	226	26.9
≥ 65	224	26.6
Age at menarche (years)		
< 12	106	12.6
12	166	19.7
13	190	22.6
≥ 14	193	23.0
*Missing*	*186*	*22.1*
Parity		
Parous	567	67.4
Nulliparous	117	13.9
*Missing*	*157*	*18.7*
Menopausal status		
Pre-menopause	255	30.3
Post-menopause	501	59.6
*Missing*	*85*	*10.1*
Tumor histotype		
Invasive ductal carcinoma	625	74.3
Lobular carcinoma	65	7.7
Medullary carcinoma	29	3.4
Apocrine carcinoma	20	2.4
Pleomorphic carcinoma	14	1.7
Metaplastic carcinoma	12	1.4
Others	29	3.4
*Missing*	*47*	*5.6*
Histologic grade		
1	11	1.3
2	169	20.1
3	600	71.3
*Missing*	*61*	*7.3*
Tumor size (T)		
Tx	15	1.8
T1	309	36.7
T2	364	43.3
T3	55	6.5
T4	43	5.1
*Missing*	*55*	*6.5*
Pathological lymph nodes (pN)		
pNx	26	3.1
pN0	439	52.2
pN1	185	22.0
pN2	88	10.5
pN3	46	5.6
*Missing*	*57*	*6.8*
Distant metastases (M)		
M0	747	88.8
M1	10	1.2
*Missing*	*84*	*10.0*
Tumor stage		
I	211	25.1
II	359	42.7
III	161	19.1
IV	10	1.2
*Missing*	*100*	11.9
Tumor infiltrating lymphocytes		
No	497	59.1
Yes	283	33.7
*Missing*	61	7.2
Lymphovascular invasion		
No	587	69.8
Yes	193	23.0
*Missing*	*61*	7.2
Necrosis		
No	480	57.1
Yes	301	35.8
*Missing*	*60*	7.1
Ki-67 (%)		
0–15	112	13.3
16–25	95	11.3
26–35	122	14.5
36–45	100	11.9
≥ 46	376	44.7
*Missing*	*36*	*4.3*
Type of surgery		
No surgery	6	0.7
Quadrantectomy	437	52.0
Mastectomy	319	37.9
*Missing*	*79*	*9.4*
Radiotherapy on residual breast^a^		
No	0	0.0
Yes	411	94.1
*Missing*	*26*	*5.9*
Post-mastectomy radiotherapy^b^		
No	236	74.0
Yes	24	7.5
*Missing*	*59*	*18.5*
Chemotherapy		
Neoadjuvant, only	34	4.0
Adjuvant, only	545	64.8
Neoadjuvant and adjuvant	73	8.7
*Missing*	*189*	*22.5*

^a^For patients who had a quadrantectomy. ^b^For patients who had a mastectomy

Table 2 shows the multivariate HRs for mortality according to selected clinical and pathological characteristics of TNBCs. After allowance for study center and age at diagnosis, all the components of the TNM staging system were significant independent prognostic factors for TNBC. In particular, compared to T1, HRs for mortality were 2.71 (95% CI 1.74–4.23) for T2, 2.85 (95% CI 1.46–5.55) for T3, and 8.13 (95% CI 4.44–14.9) for T4. Compared to pN0, HRs were 2.63 (95% CI 1.69–4.10) for pN1, 3.54 (95% CI 2.06–6.06) for pN2, and 6.10 (95% CI 3.44–10.8) for pN3. Compared to patients without metastasis at diagnosis, the HR was 6.01 (95% CI 2.72–13.3) for those with metastasis. Compared to tumor stage I, HR was 3.09 (95% CI 1.59–6.00) for stage II, 9.68 (95% CI 5.02–18.7) for stage III, and 19.8 (95% CI 7.54–51.9) for stage IV. Presence of LVI and necrosis was also significantly associated with increased mortality (HR: 2.45, 95% CI 1.71–3.51, and 1.58, 95% CI 1.11–2.24, respectively). Expression of Ki-67 over 16% were associated with increased mortality, although in the absence of a clear trend with increasing expression (HR: 1.77, 95% CI 1.08–2.91, for Ki-67 ≥ 16% compared to 0–15%). Patients with tumor histotypes other than ductal invasive or lobular carcinomas had a more favorable, though not significant, prognosis as compared to ductal invasive carcinoma (HR: 0.56, 95% CI 0.30–1.02). No significant associations were found for histologic grade (HR: 1.12, 95% CI 0.77–1.63, for grade 3 vs grade 1–2), and presence of TIL (HR: 1.24, 95% CI 0.85–1.80). For most clinical and pathological characteristics, the results were consistent when models were further adjusted for TNM-T, TNM-N, TNM-M, LVI, necrosis, and Ki-67. Only the presence of LVI was no more significantly associated to increased mortality after accounting for those clinicopathological factors (HR: 1.49, 95% CI 0.93–2.38).

**Table 2:** Hazard ratios (HRs) of mortality, and corresponding 95% confidence intervals (CIs), according to selected clinical and pathological characteristics, among 841 triple-negative breast cancers (TNBCs). Sardinia, Italy 1994–2015

		Number of deaths (%)	HR^a^ (95% CI)	HR^b^ (95% CI)
Tumor histotype				
Ductal invasive carcinoma	625	106 (16.9)	1.00^c^	1.00^c^
Lobular carcinoma	65	14 (21.2)	1.04 (0.59–1.84)	0.66 (0.31–1.42)
Other carcinomas^d^	104	12 (11.9)	0.56 (0.30–1.02)	**0.40 (0.21–0.76)**
Histologic grade				
1,2	180	40 (22.2)	1.00^c^	1.00^c^
3	600	102 (17.0)	1.12 (0.77–1.63)	0.96 (0.58–1.58)
Tumor size (T)				
T1	309	30 (9.7)	1.00^c^	1.00^c^
T2	364	67 (18.4)	**2.71 (1.74–4.23)**	**2.41 (1.40–4.15)**
T3	55	13 (23.6)	**2.85 (1.46–5.55)**	**2.24 (1.00–5.06)**
T4	43	17 (39.5)	**8.13 (4.44–14.9)**	**5.13 (2.21–11.9)**
Pathological lymph nodes (pN)				
pN0	439	39 (8.9)	1.00^c^	1.00^c^
pN1	185	42 (22.7)	**2.63 (1.69–4.10)**	**2.04 (1.22–3.40)**
pN2	88	21 (23.9)	**3.54 (2.06–6.06)**	**3.11 (1.70–5.68)**
pN3	46	19 (41.3)	**6.10 (3.44–10.8)**	**3.18 (1.51–6.71)**
Distant metastases (M)				
M0	747	117 (15.7)	1.00^c^	1.00^c^
M1	10	7 (70.0)	**6.01 (2.72–13.3)**	**5.13 (1.69–15.6)**
Tumor stage^e^				
I	211	11 (5.2)	1.00^c^	1.00^c^
II	359	48 (13.4)	**3.09 (1.59–6.00)**	**3.13 (1.56–6.27)**
III	161	52 (32.3)	**9.68 (5.02–18.7)**	**9.65 (4.66–20.0)**
IV	10	7 (70.0)	**19.8 (7.54–51.9)**	**29.0 (9.65–86.9)**
Tumor infiltrating lymphocytes (TIL)				
No	497	90 (18.1)	1.00^c^	1.00^c^
Yes	283	40 (14.1)	1.24 (0.85–1.80)	1.20 (0.76–1.91)
Lymphovascular invasion (LVI)				
No	587	82 (14.0)	1.00^c^	1.00^c^
Yes	193	48 (24.9)	**2.45 (1.71–3.51)**	1.49 (0.93–2.38)
Necrosis				
No	480	71 (14.8)	1.00^c^	1.00^c^
Yes	301	60 (19.9)	**1.58 (1.11–2.24)**	**1.61 (1.03–2.51)**
Ki-67 (%)				
0–15	112	19 (17.0)	1.00^c^	1.00^c^
16–25	95	31 (32.6)	**2.15 (1.21–3.83)**	**2.19 (1.03–4.66)**
26–35	122	24 (19.7)	1.45 (0.79–2.65)	1.65 (0.71–3.84)
36–45	*100*	18 (18.0)	1.46 (0.76–2.80)	1.69 (0.70–4.09)
≥ 46	376	57 (15.2)	**2.00 (1.14–3.51)**	**2.37 (1.08–5.21)**

^a^Estimates from multivariate proportional hazard regression models adjusted for study center and age at diagnosis. Estimates in bold are those significant at the 0.05 level. ^b^Estimates further adjusted for TNM-T, TNM-N, TNM-M, necrosis, LVI, and Ki-67. ^c^Reference category. ^d^Including medullary, apocrine, pleomorphic, and metaplastic carcinomas. ^e^Estimates not adjusted for TNM-T, TNM-N, and TNM-M

The multivariate HRs for cancer recurrence according to selected clinical and pathological characteristics were consistent with those observed for mortality, with the exception of Ki-67 and necrosis which were not found to be a significant predictor of recurrence in TNBC patients, particularly when taking into account for other clinicopathological factors (Additional file 1: Table S1).

When we considered the role of pathological lymph nodes stage and lymph node ratio on mortality among TNBC patients with positive lymph nodes (Table 3), we found that the HR for pN3 versus pN1 was 2.18 (95% CI 1.23–3.84) and that for lymph node ratio > 0.65 versus <0.20 was 3.62 (95% CI 1.96–6.68). Moreover, when we took into account for other clinicopathological factors, as well as for pathological lymph nodes stage and lymph node ratio simultaneously, the HRs became 0.80 (95% CI 0.34–1.87) and 3.05 (95% CI 1.35–6.87) for pN3 and lymph node ratio > 0.65, respectively. Consistent results were found for cancer recurrence (Additional file 1: Table S2).

**Table 3:** Hazard ratios (HRs) of mortality, and corresponding 95% confidence intervals (CIs), according to pathological lymph nodes and lymph node ratio among 319 triple-negative breast cancers (TNBCs) with positive lymph nodes. Sardinia, Italy 1994–2015

		Number of deaths (%)	HR^a^ (95% CI)	HR^b^ (95% CI)
Pathological lymph nodes (pN)				
pN1	185	42 (22.7)	1.00^c^	1.00^c^
pN2	88	21 (23.9)	1.37 (0.80–2.37)	1.13 (0.58–2.17)
pN3	46	19 (41.3)	**2.18 (1.23–3.84)**	0.80 (0.34–1.87)
Lymph node ratio				
< 0.20	169	25 (14.8)	1.00^c^	1.00^c^
0.21–0.65	93	29 (31.2)	**2.47 (1.43–4.25)**	**2.44 (1.25–4.78)**
> 0.65	48	22 (45.8)	**3.62 (1.96–6.68)**	**3.05 (1.35–6.87)**
*Missing*	*9*			

^a^Estimates from multivariate proportional hazard regression models adjusted for study center and age at diagnosis. Estimates in bold are those significant at the 0.05 level. ^b^Estimates further adjusted for TNM-T, TNM-N, TNM-M, necrosis, LVI, and Ki-67. ^c^Reference category

Using the missing indicator method to treat missing data in the multivariable models as a sensitivity analysis, we found HR estimates consistent with those presented above (data not shown).

**Fig. 1:**
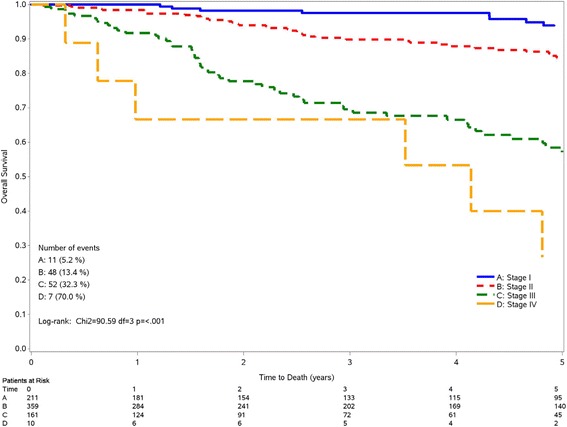
Kaplan-Meir curves for overall survival according to tumor stage among 841 triple-negative breast cancer patients. Sardinia, Italy 1994–2005

Figure 1 shows the Kaplan-Meier curves for the association between tumor stage and OS. There was a clear and significant (*p* < 0.001) reduction in OS according to increasing stage at diagnosis of TNBC. 5-years OS was 93.9% for stage I, 84.5% for stage II, 57.2% for stage III, and 26.7% for stage IV. Results were similar for DFS (Additional file 1: Figure S1).

**Fig. 2:**
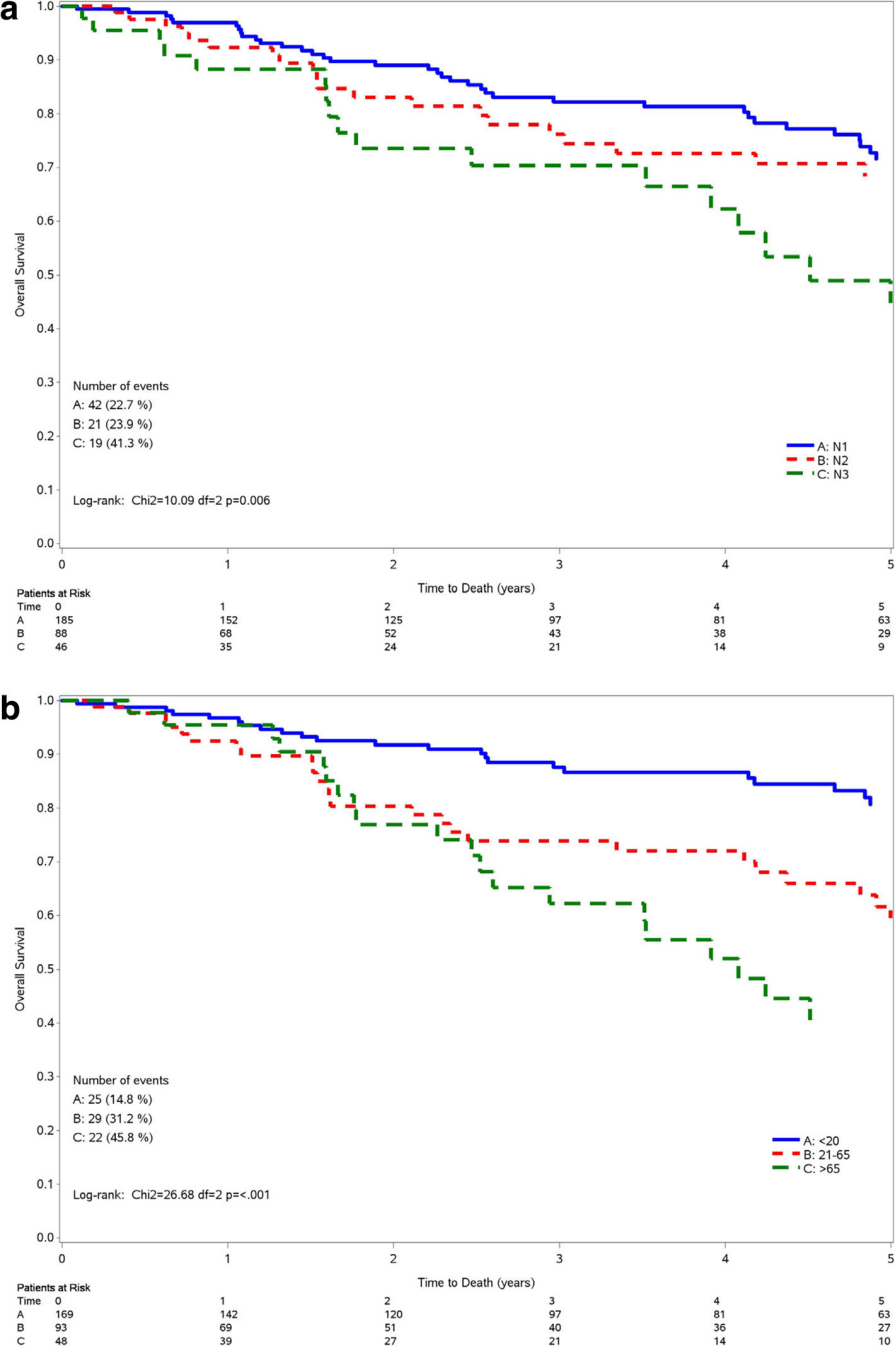
Kaplan-Meir curves for overall survival according to pathological lymph nodes stage (**a**) and lymph node ratio (**b**) among 319 triple-negative breast cancer patients with positive lymph nodes. Sardinia, Italy 1994–2005

Figure 2 shows the Kaplan-Meier curve for OS according to pathological lymph nodes stage (Fig. 1a) and lymph node ratio (Fig. 1b), among patients with positive lymph nodes. For pathological lymph nodes, the survival curves for pN1 and pN2 stage patients overlapped, while pN3 stage patients had a worse survival (*p* = 0.006). The 5-yrs survival was 71.6%, 68.3%, and 44.1% for pN1, pN2, and pN3, respectively. When considering lymph node ratio, there was significant reduction in OS according to increasing level of lymph node ratio (*p* < 0.001), 5-yrs survival being 80.7%, 59.4%, and 40.5% for <0.21, 0.21–0.65, and >0.65, respectively. Again, consistent results were found for DFS (Additional file 1: Figure S2).

## Discussion

In this uniquely large cohort of TNBC patients, we found that a high tumor stage at diagnosis – as defined by tumor size, pathological lymph nodes, and presence of metastasis – is the most important prognostic factor for cancer progression and mortality. Among other tumor features, necrosis and Ki-67 are also independently associated with increased mortality. Moreover, among lymph node positive tumors, lymph node ratio appears to be a better predictor of mortality than pathological lymph nodes.

The TNM staging system has long been identified as one of the best predictors of long-term survival and an indicator for therapeutic decisions in patients with breast cancer [22]. However, the usefulness of TNM as a prognostic factor for biologically different subtypes of breast cancer, including TNBCs, has been questioned [7, 8]. Indeed, a cohort based on 391 TNBC patients from the Samsung Medical Center, Korea, found no association between tumor stage and recurrence-free survival among TNBC patients with stage 1 to 3A, suggesting that the TNM staging system might not be a good predictor of survival outcomes in TNBC patients [8]. Moreover, a large study from the US suggested that survival in TNBC patients was affected by the presence of positive lymph nodes, but were not greatly influenced by the number of positive lymph nodes [23]. Nevertheless, a few studies found that tumor size and lymph node status have a significant association with both DFS and OS in TNBC patients [24–27]. Consistently, in our study we found a highly significant association between TNM stage and cancer progression or mortality among TNBC patients. Among single TNM staging components, both tumor size and involvement of lymph nodes were independently and significantly associated with recurrence and overall mortality. Moreover, we found that the presence of metastasis at diagnosis increased cancer recurrence and mortality by more than five-fold. Another study also showed that that patients with metastatic TNBC have poorer prognosis as compared to non-metastatic ones [28].

Increasing evidence has shown that the lymph node ratio – which takes into account not only the number of pathological lymph nodes involved but also the number of lymph nodes evaluated – is a more accurate prognostic factor for breast cancer as compared to number of lymph nodes involved [9]. A few studies have also shown that lymph node ratio is an additional independent prognostic factor to the traditional pN stage in the OS and DFS of TNBCs [27, 29, 30]. Consistently, we found that in lymph node positive patients, lymph node ratio appeared to be a stronger predictor of mortality than pN stage, allowing a better discrimination of TNBC patients at high or low risk of mortality.

Many investigations have suggested that the proliferation marker Ki-67 is a valuable prognostic marker in early breast cancer [31]. The prognostic significance of Ki-67 in TNBC patients has also been investigated in several studies providing, however, inconsistent results [24, 32–36]. Some studies did not find any association between Ki-67 and survival outcomes for TNBCs [24, 33, 35], while other larger studies showed that a relatively high Ki-67 expression (≥10%) was inversely associated with TNBC outcomes [32, 34, 36]. These results are in agreement with our findings, showing that TNBC patients with Ki-67 expression over 16% have a poorer prognosis, although the mortality did not increase with increasing expression of Ki-67. The inconsistencies of the results across various studies can be explained either by lack of analytical validity and standardization of this marker or by the use of different cut-off points for the definition of positivity to ki-67 [37]. Given these drawbacks, the use of ki-67 in the clinical practice for patients with TNBCs, as other breast cancers, remains still debatable [11, 31].

Limited information is available on the association between histological subtype of TNBC and survival outcomes. One study conducted on 476 TNBC patients from Belgium suggested some differences in DFS according to tumor histology. However, given the relatively low number of TNBC histotypes other than invasive ductal carcinoma, the study was not able to provide significant estimates [38]. A larger study conducted on 781 TNBC patients from Italy found that, compared with patients with invasive ductal carcinoma, OS and DFS were less favorable in women with metaplastic carcinoma, and more favorable in those with adenoid cystic and medullary subtypes, while no difference was observed for lobular carcinoma [12]. Accordingly, we did not find any difference in terms of recurrence and mortality between lobular and invasive ductal carcinomas, whereas other TNBC subtypes (mostly medullary and apocrine carcinomas) showed significantly better outcomes than invasive ductal carcinomas.

Among the best-established prognostic factors for breast cancer there is histologic grade [10]. This notwithstanding, in our large cohort of TNBC patients – as reported in a few other smaller studies [8, 26] – grade had no role in survival outcomes. This may be at least in part due to the high histological grade of TNBC patients [3], which might make it difficult to disentangle the role of grade on TNBC prognosis. Indeed, in our cohort only 1.5% of patients were G1 and over 3 out of 4 patients were G3.

Various large studies recently found that tumor TILs (mainly stromal) – a surrogate marker of adaptive immune response – is associated with a favorable prognosis in TNBC patients [39–42], although the use of TILs as an additional prognostic factors in TNBCs is not yet recommend giving the lack of standardization and clinical validation of this marker [13]. In our cohort, although death rates were lower among TNBC patients with TILs, no significant association was found between TILs and cancer progression or mortality. However, we had no information on the number/proportion of TILs and we did not specifically measured stromal TILs.

LVI – which refers to the invasion of lymphatic spaces and blood vessels – has long been considered a relevant prognostic marker of breast cancer, although it has not been incorporated in most internationally recognized staging system as the AJCC/TMN one [14, 21]. A few small studies which have investigated the relationship between LVI and DFS or OS in patients with TNBC showed that LVI is an independent predictor of poor outcome [14, 26, 43]. In our large cohort we found that LVI presence has a negative impact on both tumor recurrence and mortality when taking into account only study center and age at diagnosis. However, after allowance for other clinicopathological characteristics, the association between LVI and mortality was no more significant, thus do not supporting a relevant prognostic role of this marker.

Scanty data are available on the role of necrosis on prognostic outcomes in TNBC patients. In a study on 154 TBNCs from China, tumor necrosis was found to be a significant prognostic factor, although only results from univariate analyses were provided [15]. In our large cohort, necrosis at baseline was significantly associated to survival outcomes, even after allowance for other clinicopathological factors.

The results of this study should be interpreted after taking into consideration various limitations, mainly inherent to its retrospective design. Thus, we could not retrieve information on vital status at follow-up for 311 out of 1152 TNBC patients (about 27%) and we had to exclude them from the present analyses. Moreover, for some patients important clinical and pathological data were missing because not originally included in the medical records, and those missing information may have to some extent influenced the associations evaluated. Furthermore, some misclassification of patients may have resulted from the classification of tumors in different laboratories across hospital centers, where clinical and pathological testing practices can vary. However, pathology materials were reviewed centrally by three pathologists following the same national/international breast cancer guidelines in order to uniformely classify TNBCs across hospital centers and standardize ER, PgR and HER2 immunohistochemical results for TNBC samples, according to the ASCO/CAP recommendations [17].

The strengths of our study include its uniquely large sample size – including from one third up to half of all new Sardinian TNBC patients over the study period, the comprehensive and standardized nature of the registry database with patients’ characteristics, pathological tumor features, cancer treatments, and the complete ascertainment of patient status at regular follow-up intervals. This also allowed us to derive multivariate HR estimates for OS and DFS after allowance for a number of potential confounders.

## Conclusions

In this uniquely large cohort, we provide further evidence that, besides tumor stage at diagnosis, lymph node ratio among lymph node positive tumors is an additional relevant predictor of mortality and recurrence in TNBCs, while Ki-67 seems to be predictive of mortality, but not of recurrence.

## Additional files

Additional file 1: Table S1. Hazard ratios (HRs) of recurrence, and corresponding 95% confidence intervals (CIs), according to selected clinical and pathological characteristics, among 825 triple-negative breast cancers (TNBCs). Sardinia, Italy 1994-2015. **Table S2.** Hazard ratios (HRs) of recurrence, and corresponding 95% confidence intervals (CIs), according to pathological lymph nodes and lymph node ratio among 311 triple-negative breast cancers (TNBCs) with positive lymph nodes. Sardinia, Italy 1994-2015. **Figure S1.** Kaplan-Meir curves for disease-free survival according to tumor stage among 825 triple-negative breast cancer patients. Sardinia, Italy 1994–2005. **Figure S2.** Kaplan-Meir curves for disease-free survival according to pathological lymph nodes stage (a) and lymph node ratio (b) among 311 triple-negative breast cancer patients with positive lymph nodes. Sardinia, Italy 1994–2005 (DOC 658 kb)

## Abbreviations

AJCC: American Joint Committee on Cancer criteria
CI: confidence interval
DFS: Disease-free survival
ER: estrogen receptor
HER2: human epidermal growth factor receptor 2
HR: hazard ratio
LVI: lymphovascular invasion
M: metastasis
OS: Overall survival
pN: pathological N stage
PR: progesterone receptor
SD: standard deviation
T: tumor stage
TIL: tumor infiltrating lymphocytes
TNBC: triple-negative breast cancer
