# Supplementary material for: Clinical and pathological factors influencing survival in a large cohort of triple-negative breast cancer patients
Source: BMC Cancer. 2018 Jan 8;18:56. doi: 10.1186/s12885-017-3969-y (PMC5759886; doi:10.1186/s12885-017-3969-y)

**Supplementary Table 1**: Hazard ratios (HRs) of recurrence, and corresponding 95% confidence intervals (CIs), according to selected clinical and pathological characteristics, among 825 triple-negative breast cancers (TNBCs). Sardinia, Italy 1994-2015.

|  | **TNBC (N)** | **Number of recurrences (%)** | **HR^a^ (95% CI)** | **HR^b^ (95% CI)** |
| --- | --- | --- | --- | --- |
| Tumor histotype |  |  |  |  |
| Ductal invasive carcinoma | 614 | 187 (30.5) | 1.00^c^ | 1.00^c^ |
| Lobular carcinoma | 64 | 23 (35.9) | 0.96 (0.62-1.49) | 0.95 (0.57-1.58) |
| Other carcinomas^d^ | 100 | 28 (28.0) | 0.72 (0.48-1.08) | **0.54 (0.35-0.84)** |
| Histologic grade |  |  |  |  |
| 1,2 | 176 | 62 (35.2) | 1.00^c^ | 1.00^c^ |
| 3 | 589 | 180 (30.6) | 1.14 (0.85-1.54) | 1.17 (0.81-1.69) |
| Tumor size (T) |  |  |  |  |
| T1 | 301 | 65 (21.6) | 1.00^c^ | 1.00^c^ |
| T2 | 359 | 113 (31.5) | **1.78 (1.30-2.44)** | **1.82 (1.28-2.60)** |
| T3 | 55 | 25 (45.5) | **2.94 (1.83-4.73)** | **2.27 (1.32-3.89)** |
| T4 | 41 | 25 (61.0) | **5.72 (3.57-9.18)** | **4.53 (2.53-8.09)** |
| Pathological lymph node status (pN) |  |  |  |  |
| pN0 | 433 | 93 (21.5) | 1.00^c^ | 1.00^c^ |
| pN1 | 180 | 65 (36.1) | **1.84 (1.34-2.54)** | **1.53 (1.07-2.19)** |
| pN2 | 86 | 36 (41.9) | **2.50 (1.69-3.69)** | **2.23 (1.47-3.39)** |
| pN3 | 45 | 26 (57.8) | **4.41 (2.79-6.98)** | **3.45 (2.02-5.91)** |
| Tumor stage^e^ |  |  |  |  |
| I | 208 | 35 (16.8) | 1.00^c^ | 1.00^c^ |
| II | 358 | 97 (27.1) | **1.83 (1.24-2.71)** | **1.89 (1.25-2.87)** |
| III | 160 | 79 (49.4) | **4.44 (2.97-6.65)** | **4.70 (3.03-7.30)** |
| Tumor infiltrating lymphocytes (TIL) |  |  |  |  |
| No | 487 | 161 (33.1) | 1.00^c^ | 1.00^c^ |
| Yes | 278 | 70 (25.2) | 1.23 (0.92-1.63) | 1.17 (0.84-1.64) |
| Lymphovascular invasion (LVI) |  |  |  |  |
| No | 577 | 163 (28.2) | 1.00^c^ | 1.00^c^ |
| Yes | 188 | 68 (36.2) | **1.73 (1.30-2.31)** | 1.09 (0.77-1.55) |
| Necrosis |  |  |  |  |
| No | 469 | 135 (28.8) | 1.00^c^ | 1.00^c^ |
| Yes | 297 | 97 (32.7) | **1.33 (1.03-1.74)** | 1.27 (0.93-1.73) |
| Ki-67 (%) |  |  |  |  |
| 0-15 | 110 | 36 (32.7) | 1.00^c^ | 1.00^c^ |
| 16-25 | 95 | 46 (48.4) | 1.53 (0.98-2.38) | 1.32 (0.79-2.20) |
| 26-35 | 120 | 43 (35.8) | 1.28 (0.82-2.00) | 1.27 (0.75-2.14) |
| 36-45 | 97 | 29 (29.9) | 1.17 (0.71-1.92) | 1.10 (0.62-1.94) |
| ≥ 46 | 368 | 99 (26.9) | 1.32 (0.88-1.99) | 1.25 (0.77-2.02) |

**^a^** Estimates from multivariate proportional hazard regression models adjusted for study center and age at diagnosis. Estimates in bold are those significant at the 0.05 level. **^b^** Estimates further adjusted for TNM-T, TNM-N, necrosis, LVI, and Ki-67. **^c^** Reference category. ^d^ Including medullary, apocrine, pleomorphic, and metaplastic carcinomas. ^e^ Estimates not adjusted for TNM-T, and TNM-N.

**Supplementary Table 2**: Hazard ratios (HRs) of recurrence, and corresponding 95% confidence intervals (CIs), according to pathological lymph nodes and lymph node ratio among 311 triple-negative breast cancers (TNBCs) with positive lymph nodes. Sardinia, Italy 1994-2015.

|  | **TNBC (N)** | **Number of recurrences (%)** | **HR^a^ (95% CI)** | **HR^b^ (95% CI)** |
| --- | --- | --- | --- | --- |
|  |  |  |  |  |
| Pathological lymph nodes (pN) |  |  |  |  |
| pN1 | 180 | 65 (36.1) | 1.00^c^ | 1.00^c^ |
| pN2 | 86 | 36 (41.9) | 1.38 (0.91-2.10) | 1.31 (0.79-2.17) |
| pN3 | 45 | 26 (57.8) | **2.28 (1.42-3.68)** | 1.45 (0.73-2.88) |
| Lymph node ratio |  |  |  |  |
| <0.20 | 165 | 51 (30.9) | 1.00^c^ | 1.00^c^ |
| 0.21-0.65 | 92 | 39 (42.4) | **1.65 (1.08-2.52)** | 1.38 (0.82-2.32) |
| >0.65 | 47 | 31 (66.0) | **2.98 (1.84-4.82)** | **2.02 (1.05-3.88)** |
| *Missing* | *7* |  |  |  |
|  |  |  |  |  |

**^a^** Estimates from multivariate proportional hazard regression models adjusted for study center and age at diagnosis. Estimates in bold are those significant at the 0.05 level. **^b^** Estimates further adjusted for TNM-T, TNM-N, necrosis, LVI, and Ki-67. **^c^** Reference category.

**Supplementary Figure 1**


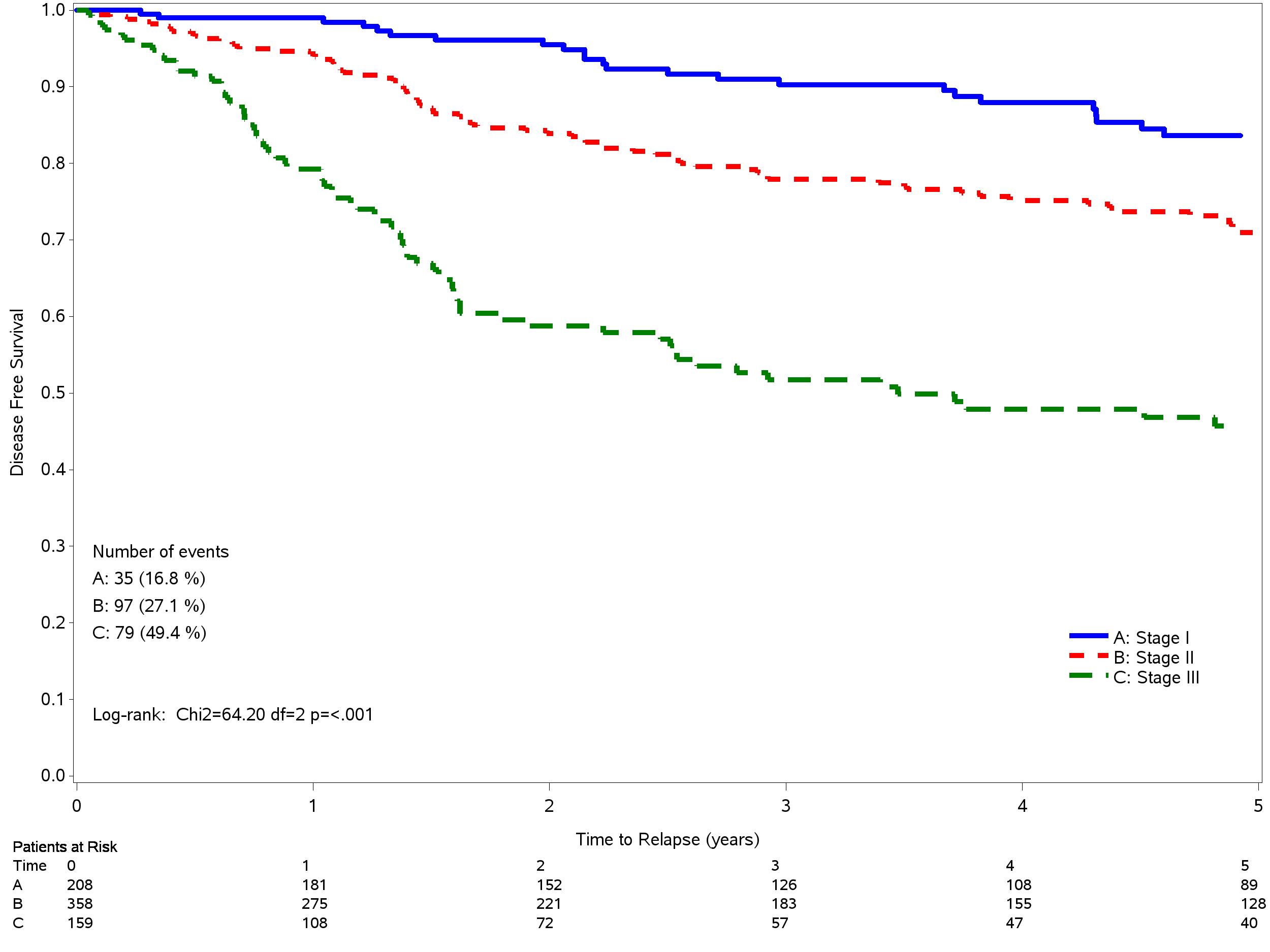


**Supplementary Figure 2**

**A**


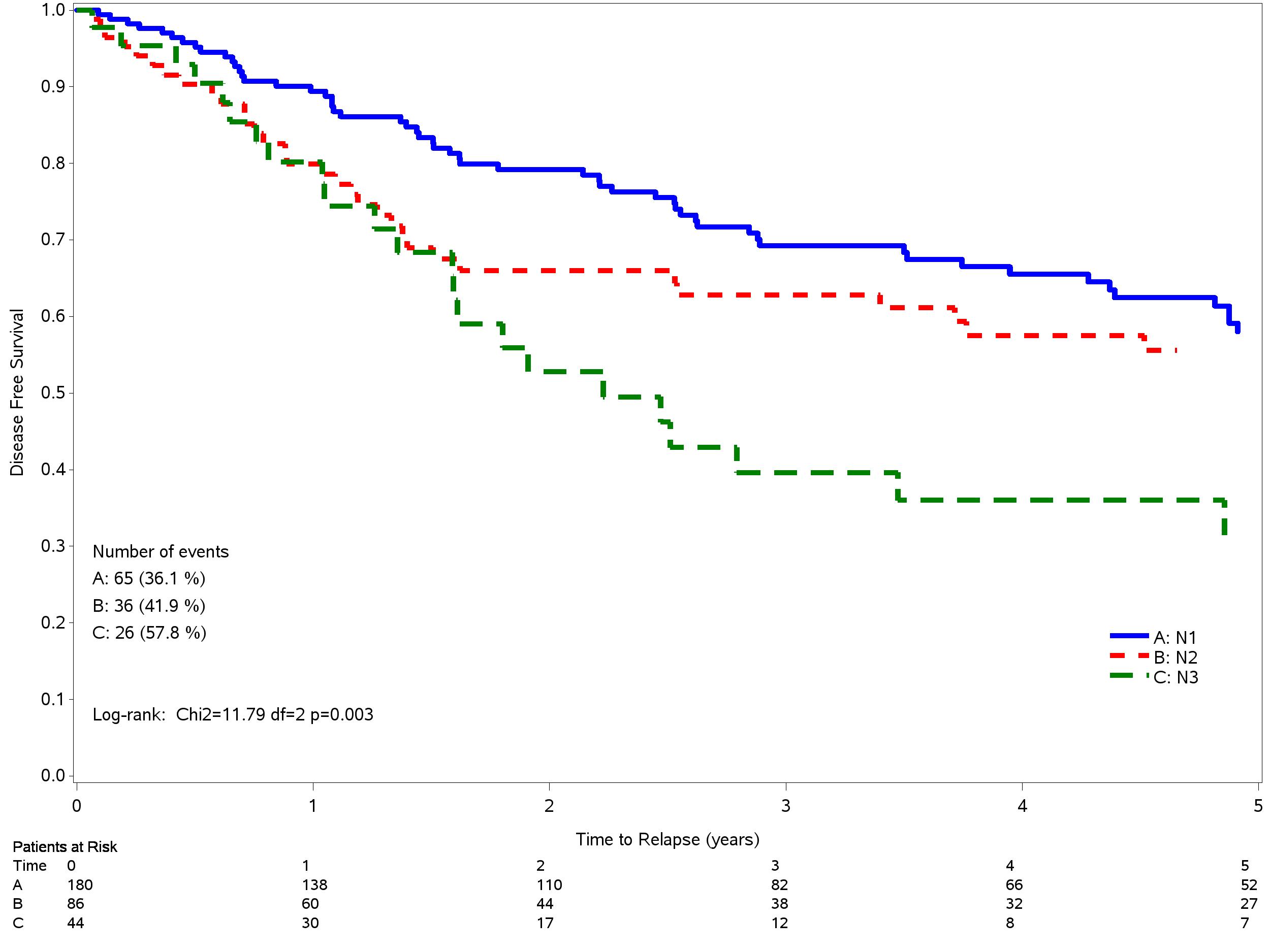


**B**


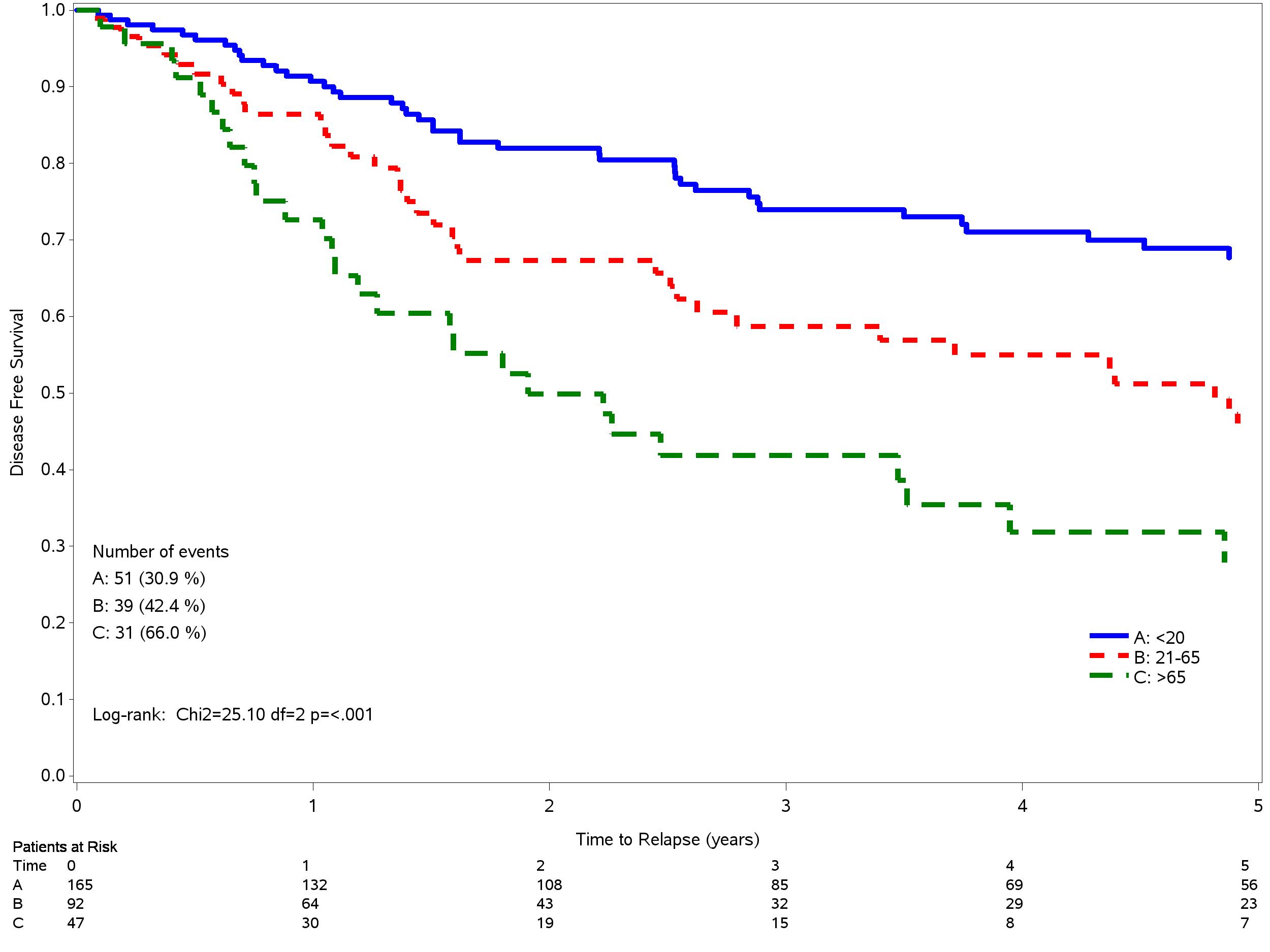

Supplement: Supplementary file 1 — Hazard ratios (HRs) of recurrence, and corresponding 95% confidence intervals (CIs), according to selected clinical and pathological characteristics, among 825 triple-negative breast cancers (TNBCs). Sardinia, Italy 1994-2015. Table S2. Hazard ratios (HRs) of recurrence, and corresponding 95% confidence intervals (CIs), according to pathological lymph nodes and lymph node ratio among 311 triple-negative breast cancers (TNBCs) with positive lymph nodes. Sardinia, Italy 1994-2015. Figure S1. Kaplan-Meir curves for disease-free survival according to tumor stage among 825 triple-negative breast cancer patients. Sardinia, Italy 1994–2005. Figure S2. Kaplan-Meir curves for disease-free survival according to pathological lymph nodes stage (a) and lymph node ratio (b) among 311 triple-negative breast cancer patients with positive lymph nodes. Sardinia, Italy 1994–2005 (DOC 658 kb) [file 12885_2017_3969_MOESM1_ESM.doc]
